# Perceived walking difficulties in Parkinson’s disease – predictors and changes over time

**DOI:** 10.1186/s12877-021-02113-0

**Published:** 2021-04-01

**Authors:** Magnus Lindh-Rengifo, Stina B. Jonasson, Susann Ullén, Niklas Mattsson-Carlgren, Maria H. Nilsson

**Affiliations:** 1 Department of Health Sciences, Faculty of Medicine, Lund University, Lund, Sweden; 2 Memory Clinic, Skåne University Hospital, Malmö, Sweden; 3 Clinical Studies Sweden – Forum South, Skåne University Hospital, Lund, Sweden; 4 Clinical Memory Research Unit, Department of Clinical Sciences Malmö, Lund University, Lund, Sweden; 5 Department of Neurology, Skåne University Hospital, Lund, Sweden; 6 Wallenberg Centre for Molecular Medicine, Lund University, Lund, Sweden

**Keywords:** Perceived walking difficulties, Parkinson disease, Mobility limitation, Prediction, Multivariable regression

## Abstract

**Background:**

People with Parkinson’s disease (PD) have described their walking difficulties as linked to activity avoidance, social isolation, reduced independence and quality of life. There is a knowledge gap regarding predictive factors of perceived walking difficulties in people with PD. Such knowledge could be useful when designing intervention studies. This study aimed to investigate how perceived walking difficulties evolve over a 3-year period in people with PD. A specific aim was to identify predictive factors of perceived walking difficulties.

**Methods:**

One hundred forty-eight people with PD (mean age 67.9 years) completed the Generic Walk-12 (Walk-12G) questionnaire (which assesses perceived walking difficulties) at both baseline and the 3-year follow-up. Paired samples t-test was used for comparing baseline and follow-up mean scores. Multivariable linear regression analyses were used to identify predictive factors of perceived walking difficulties.

**Results:**

Perceived walking difficulties increased after 3 years: mean Walk-12G score 14.8 versus 18.7, *p* < 0.001. Concerns about falling was the strongest predictor (β = 0.445) of perceived walking difficulties, followed by perceived balance problems while dual tasking (β = 0.268) and pain (β = 0.153). Perceived balance problems while dual tasking was the strongest predictor (β = 0.180) of a change in perceived walking difficulties, followed by global cognitive functioning (β = − 0.107).

**Conclusions:**

Perceived walking difficulties increase over time in people with PD. Both personal factors (i.e. concerns about falling) and motor aspects (i.e. balance problems while dual tasking) seem to have a predictive role. Importantly, our study indicates that also non-motor symptoms (e.g. pain and cognitive functioning) seem to be of importance for future perceived walking difficulties. Future intervention studies that address these factors need to confirm their preventative effect on perceived walking difficulties.

## Background

Walking difficulties are common in people with Parkinson’s disease (PD) [1] and are associated with a loss of independence [2], activity limitations [3, 4], falls [5] as well as decreased social participation [6] and quality of life [7]. Gait patterns change at an early stage of PD [8], and increases further if combined with dual tasking [9], i.e. simultaneous performance of a cognitive or motor task while walking. All considered, this makes it important to assess and address walking difficulties in PD.

There is a difference in walking performance when tested in a clinical setting, where walking conditions are often optimized, as compared to daily life situations where spaces might be narrow, dimly lit, etc. This can render better objective gait performance in a clinical setting than would be the case in another context [10, 11]. Comparing objective gait measures with measures of perceived walking difficulties has shown weak to moderate associations [12], indicating that they are not interchangeable constructs. As such, measures of perceived walking difficulties can serve as a complement to clinical walking tests, providing the patients perspective.

An increased knowledge of factors that predict objective as well as perceived walking difficulties could nurture the development of future preventative and rehabilitative approaches. Cross-sectional studies have found that older age [13–15], lower cognitive functioning [13], reduced balance [13], lower balance confidence [13, 15], fear of falling [14], fall history [14], mood disorder [14] and worse disease severity [14, 15] are associated with objective gait impairments. Less is known about predictive factors of objective and perceived walking difficulties.

Qualitative studies that involved people with PD have described that walking difficulties relate to both motor and non-motor factors, such as freezing of gait (i.e. FOG), fear of falling, pain and fatigue [16, 17]. Sudden changes in their walking ability induce an uncertainty whether they will manage activities or not [6, 18, 19]. On the other hand, belief in their walking ability can serve as an enabler of activity and participation in socially meaningful activities [6].

Cross-sectional quantitative studies that addressed perceived walking difficulties in people with PD have identified several associated factors. One study found that two objective gait domains (i.e. pace and variability) were moderately associated with perceived walking difficulties [12]. Another cross-sectional study, based on the same sample as the present study, identified FOG as the strongest contributing factor, followed by general self-efficacy, fatigue, PD duration, lower extremity function, orthostatic hypotension, bradykinesia and postural instability [20]. However, a longitudinal study design is a prerequisite for identifying predictive factors; this is lacking in relation to perceived walking difficulties in people with PD.

This study aimed to investigate how perceived walking difficulties evolve over a 3-year period in people with PD. A specific aim was to identify predictive factors of perceived walking difficulties.

## Methods

This study used longitudinal data from the larger project “Home and Health in People Ageing with Parkinson’s disease” (PI: Nilsson, MH). It includes baseline and 3-year follow-up data, collected in 2013 and 2016, respectively. For a more comprehensive description of the design and methods, please read the study protocol [21].

### Participants and recruitment

In the larger project, participants were recruited (outpatient context) from three hospitals in southern Sweden. PD diagnosis (ICD10-code G20.9) since at least 1 year constituted inclusion criterion. Exclusion criteria were as follows: difficulties understanding or speaking Swedish, living outside Skåne County, severe cognitive difficulties and/or other reasons that hindered them from giving informed consent or taking part in the majority of the data collection (e.g. hallucinations or a recent stroke).

**Fig. 1:**
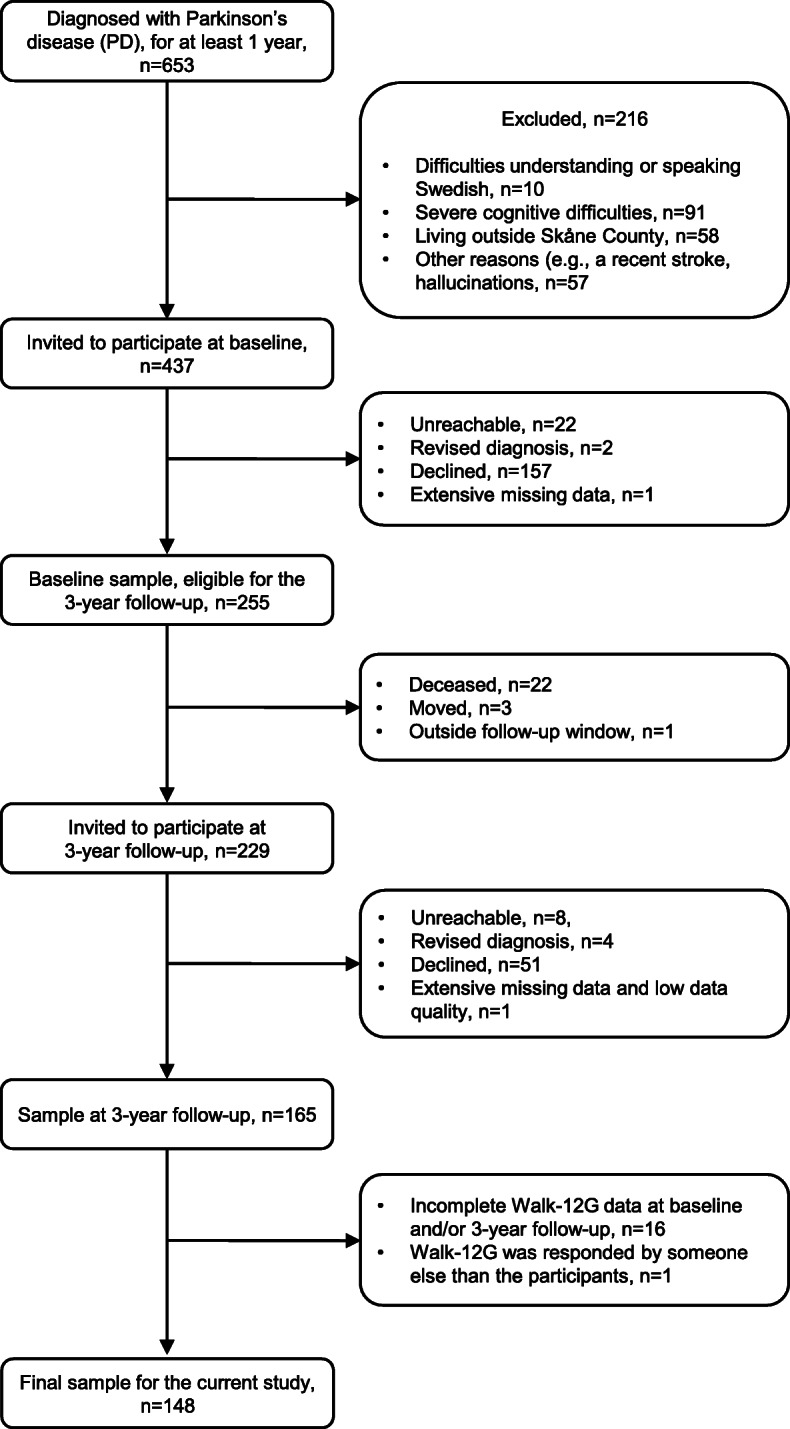
Flow chart: Participant recruitment process

The selection process was performed by the specialist PD nurse responsible for the patients at the outpatient clinic, i.e. at the respective hospital. In some instances, the PD nurse checked medical records and contacted the responsible movement disorder neurologist. Flowcharts and in-depth descriptions of the recruitment procedures are previously published for baseline [20] as well as the 3-year follow-up [22]. A flowchart (slightly revised from previous publications [20, 22]) is presented in Fig. 1.

At baseline, the final sample size was 255 participants. To be considered eligible for participation at the 3-year follow-up (± 3 months), the participants should have completed the baseline assessments and then agreed to be contacted again (*n* = 255). At the 3-year follow-up, eight were unreachable and 22 persons were deceased. One person was excluded since they responded outside the follow-up window. Out of those contacted, seven additional participants were excluded, i.e. had moved outside Skåne county or had no longer the diagnosis PD. This resulted in 217 potential participants. Out of these, 51 (23.5%) declined further participation. Extensive missing data and low data quality led to that an additional person was excluded. In total, 165 participants took part in the 3-year follow-up.

A specific inclusion criterion applied for the current study; only participants with a total score on the Generic Walk-12 (Walk-12G; assesses perceived walking difficulties) questionnaire at both baseline and the 3-year follow-up were included (*n* = 149). One additional participant was excluded due to not having completed the questionnaire themselves, nor getting help in responding, but someone else had in fact responded. This rendered a final study sample of 148 participants. At baseline, their mean (SD) age was 67.9 (±8.92) years, and 33.1% were female. Their median (q1-q3) PD duration was 8 (5–11) years. Median (q1-q3) PD severity during “on-state” according to the Hoehn & Yahr staging was 2 (2–3); possible scoring range 1–5 (higher = worse) [23]. Additional descriptive information is presented in Table 1.

**Table 1:** Participants’ characteristics at baseline and univariable linear regression analyses with Walk-12G scores at the 3-year follow-up as the dependent variable, *N* = 148

Independent variables	Descriptives	Missing	Univariable regression analyses	
		n	B (95% CI); β	*p*-value
Age (years), mean (SD)	67.9 (8.92)	–	0.530 (0.325, 0.734); 0.390	**< 0.001**
Sex (women = 1), n (%)	49 (33.1)	–	3.35 (− 0.806, 7.52); 0.131	0.113
Concerns about falling (FES-I), median (q1-q3)	23 (18–36)	2	0.733 (0.616, 0.850); 0.719	**< 0.001**
Dual task: perceived balance problems (yes = 1), n (%)	89 (60.1)	–	14.2 (10.9, 17.6); 0.576	**< 0.001**
Postural instability (UPDRS III, item 30, scores ≥1, yes = 1), n (%)	112 (75.7)	–	8.33 (3.94, 12.8); 0.296	**< 0.001**
Bradykinesia (UPDRS III, item 31, scores ≥1, yes = 1), n (%)	87 (58.8)	–	6.37 (2.49, 10.3); 0.260	**0.001**
Freezing of gait (FOGQsa, item 3, scores ≥1, yes = 1), n (%)	81 (54.7)	–	11.6 (8.15, 15.2); 0.480	**< 0.001**
Worse lower extremity function (Five chair stands test ≥16.0 s, yes = 1), n (%)^a^	76 (51.4)	–	6.55 (2.74, 10.4); 0.271	**0.001**
Orthostatism (NMSQuest, item 20, yes = 1), n (%)	73 (49.3)	–	8.12 (4.39, 11.9); 0.336	**< 0.001**
Bothered by pain (yes = 1), n (%)	93 (62.8)	–	7.62 (3.72, 11.6); 0.305	**< 0.001**
Cognitive functioning (MoCA), mean (SD)	25.7 (3.06)	2	−1.24 (− 1.86, −0.623); − 0.314	**< 0.001**
Fatigue (NHP-EN, yes = 1), n (%)	76 (51.4)	–	11.2 (7.73, 14.8); 0.465	**< 0.001**
General Self-Efficacy (GSE), mean (SD)	29.9 (6.19)	1	−0.735 (− 1.03, − 0.436); − 0.375	**< 0.001**
Depressive symptoms (GDS-15), median (q1-q3)	2 (1–4)	5	1.53 (0.851, 2.21); 0.352	**< 0.001**

Walk-12G = Generic Walk-12 (0–42, higher = worse); B = unstandardized regression coefficient; β = standardized regression coefficient; *FES-I* Falls Efficacy Scale-International (16–64, higher = worse), *UPDRS III* Unified Parkinson’s Disease Rating Scale, motor examination (item scores 0–4, higher = worse), *FOGQsa* Self-administered version of the Freezing of Gait Questionnaire (item 3 scores 0–4, higher = worse), *NMSQuest* Non-motor Symptoms Questionnaire, *MoCA* Montreal Cognitive Assessment (0–30, higher = better), *NHP-EN* Energy subscale of the Nottingham Health Profile (those who affirmed at least one out of three dichotomous questions were classified as having fatigue), *GSE* General Self-Efficacy Scale (10–40, higher = better), *GDS-15* Geriatric Depression Scale (0–15, higher = worse)

^a^12 participants did not manage the test. These were categorized as having worse lower extremity function (i.e. ≥ 16.0 s)

### General procedure

A self-administered postal survey preceded the home visit by about 10 days. The home visit included a structured interview and clinical assessments. Baseline and the 3-year follow-up were similar in terms of general procedure and data collection. We used Walk-12G data from both baseline and the 3-year follow-up. All other data in the present study were collected at baseline.

### Data collections

#### Perceived walking difficulties

Perceived walking difficulties was assessed by using the Walk-12G [24], which constituted the dependent variable in this study. Walk-12G is a self-administered questionnaire, which assesses perceived walking difficulties during the past 2 weeks. The 12 items address various aspects of perceived walking difficulties, such as perceived limitations in the ability to climb stairs, balance problems and effort level while walking. The summed total score ranges between 0 and 42 (higher = worse). The Walk-12G has been psychometrical evaluated and has shown to be both reliable and valid in people with PD [24].

#### Independent variables

Independent variables were selected based on results from prior cross-sectional PD studies, as well as theoretical and clinical reasoning regarding their relationship with perceived walking difficulties.

At the home visits, clinical assessments addressed different types of functioning, such as lower extremity function, bradykinesia, postural instability and global cognitive functioning. Lower extremity function was assessed by using the Five chair stands test (1 trial), which was performed “as fast as possible” [25]. Participants were categorized into two groups based on their median result: those who completed the Five chair stands test in 16 s or more were categorized as having worse lower extremity function (coded 1); this time limit is identical to the cut-off value for an increased fall-risk in people with PD [26]. Participants that did not manage the test (*n* = 12) were categorized as having worse lower extremity function (i.e. coded 1). Two items of the motor part of the Unified Parkinson’s Disease Rating Scale (UPDRS III) were used: postural stability (item 30) and bradykinesia (item 31) [27]. The items (originally rated 0 to 4, higher = worse) were dichotomized. A score of 0 was considered as having no problem (coded 0), whereas a score of one to four was categorized as having postural instability or bradykinesia, respectively (coded 1). Global cognitive functioning was assessed by using the Montreal Cognitive Assessment (MoCA), scored 0–30 (higher = better) [28].

The postal survey included several self-administered questions and questionnaires that addressed personal factors, different motor aspects and non-motor symptoms related to PD, such as general self-efficacy, concerns about falling, FOG and fatigue. General self-efficacy was assessed by using the General Self-Efficacy scale, scored 10–40 points (higher = better) [29]. Concerns about falling was assessed using the Falls Efficacy Scale-International (FES-I), scored 16–64 (higher = worse) [30]. For assessing FOG, the third item of the self-administered version of the Freezing of Gait Questionnaire (FOGQsa) [31] was utilized. Possible scoring range for item 3 is 0–4 (higher = worse); participants scoring > 0 were categorized as freezers [32]. Fatigue was evaluated using the Nottingham Health Profile, energy subscale (NHP-EN) [33]. Participants were classified as fatigued if they affirmed one or more out of three dichotomous questions [34]. A dichotomous (Yes/No) question addressed perceived balance problems while dual tasking: “Do you experience balance problems while standing or walking when doing more than one thing at a time, e.g. carrying a tray while walking?” [21] Item 20 of the Non-Motor Symptoms Questionnaire (NMSQuest) was used for addressing orthostatism (Yes/No) [35].

Additional data collection included pain (“Are you bothered by pain?” Yes/No) and depressive symptoms, which were assessed by using the Geriatric Depression Scale (GDS-15), scored 0–15 (higher = worse) [36]. The two latter were interview-administered at the home visit. For descriptive purposes, we also reported PD duration.

### Statistical analysis

The paired samples t-test was used to compare mean total scores of the Walk-12G, i.e. from baseline and the 3-year follow-up. For Walk-12 G scores, standard error of measurement (SEM) was calculated using the formula SEM = SDpooled × $\sqrt{1- Cronbach\ \alpha }$. SEM was calculated for baseline and follow-up scores, respectively.

Pearson’s correlation coefficients (r) were used for studying the relationship between potential independent variables in the upcoming multivariable regression analysis. There were no signs of multicollinearity between the independent variables. That is, no correlation exceeded 0.7. However, FES-I scores at baseline were strongly correlated (r_s_ = 0.869) with baseline Walk-12G scores, which were included as a controlling factor in one of the regression models.

Univariable linear regression analyses were used for studying the associations between the dependent variable (Walk-12G at the 3-year follow-up) and independent variables (Table 1). All associations fulfilled the criterion *p* < 0.3. Consequently, all independent variables were simultaneously included in the following multivariable linear regression analyses (method: enter) to avoid leaving out a confounding variable. As older age is strongly associated with walking difficulties in people with PD [14], age was included as a controlling factor.

Model 1 identifies factors that can predict perceived walking difficulties at the 3-year follow up. A second multivariable model (i.e. Model 2) was created, controlling for baseline Walk-12G scores in order to identify predictive factors of a change in Walk-12G over a 3-year period, i.e. given the Walk-12G score at baseline. In both multivariable analyses, we inspected *p*-values and estimates for each independent variable and manually removed the variable with the highest p-value from the model. This procedure continued until the *p*-value was < 0.1 for all remaining independent variables.

Residuals of all final multivariable models were visually inspected for normality, linearity and constant variance. Unadjusted and adjusted R^2^ indicate the predictive capacity of the models. Statistical significance was set to a 0.05 level. All statistical analyses were performed using SPSS statistics, version 25 (IBM Corporation, Armonk, NY, United States).

## Results

The mean (SD) Walk-12G score increased (i.e. worsened) from 14.8 (±10.8) at baseline to 18.7 (±12.1) 3 years later: mean difference 3.9, 95% confidence interval 2.6–5.2, *p* < 0.001. The SEM for Walk-12G was 2.59 at baseline, whereas it was 2.32 at the 3-year follow-up. Detailed data is presented in Table 2.

**Table 2:** Item and total scores of the Walk-12G (including Cronbach α and SEM), *N* = 148

Walk-12G items (abbreviated)	Baseline Mean (SD)	3-year follow-up Mean (SD)
1. Need to use support when walking indoors	0.62 (0.723)	0.84 (0.809)
2. Need to use support when walking outdoors	0.64 (0.849)	0.93 (0.901)
3. Limited ability to run	1.24 (0.860)	1.44 (0.827)
4. Difficult to stand when doing things	1.12 (1.15)	1.49 (1.31)
5. Limited ability to climb up and down stairs	0.97 (1.18)	1.57 (1.40)
6. Problems balancing when standing or walking	1.34 (1.15)	1.74 (1.33)
7. Limited ability to walk	1.31 (1.25)	1.70 (1.29)
8. Effortful walking	1.47 (1.20)	1.76 (1.29)
9. Smoothness of walking affected	1.49 (1.13)	1.76 (1.22)
10. Need to concentrate on walking	1.32 (1.24)	1.54 (1.30)
11. Limited walking distance	1.62 (1.40)	2.01 (1.46)
12. Slow walking	1.62 (1.15)	1.92 (1.26)
Total score, Walk-12G	14.8 (10.8)	18.7 (12.1)^†^
Internal consistency (Cronbach α)	0.949	0.959
Standard error of measurement, SEM	2.59	2.32

Walk-12G = Generic Walk-12. Possible scoring range for items 1–3: 0–2; items 4–12: 0–4, possible total scoring range 0–42, higher = worse

SEM = SD_pooled_ × $$ \sqrt{1- Cronbach\ \alpha } $$

SD_pooled_ = √ ((SD_baseline_^2^ + SD_3-year_^2^)/2)

^†^ *p* < 0.001, Paired Samples *t* Test

### Univariable regression analyses

Results from the univariable linear regression analyses are presented in Table 1. Concerns about falling had the strongest effect (based on the standardized regression coefficients, β) on perceived walking difficulties at the 3-year follow-up (β = 0.719, *p* < 0.001), followed by perceived balance problems while dual tasking (β = 0.576, *p* < 0.001).

### Multivariable regression analyses

#### Model 1 (controlled for age)

The multivariable linear regression analysis (controlled for age) resulted in a model that included five variables, and three of them did significantly predict perceived walking difficulties 3 years later. The variable with the strongest effect was concerns about falling (β = 0.445), followed by perceived balance problems while dual tasking (β = 0.268) and pain (β = 0.153). The model accounted for 61.4% of the variance (adjusted R^2^) in Walk-12G scores at the 3-year follow-up. See Table 3 for further details.

**Table 3:** Multivariable linear regression analyses with Walk-12G at 3-year follow-up as the dependent variable: Model I (controlled for age at baseline), *n* = 144

Independent variables (assessed at baseline)^a^	B (95% CI)	*P*-value	β
Concerns about falling (FES-I)	0.461 (0.325, 0.597)	**< 0.001**	0.445
Dual task: perceived balance problems (yes = 1)	6.55 (3.61, 9.49)	**< 0.001**	0.268
Bothered by pain (yes = 1)	3.79 (1.08, 6.50)	**0.006**	0.153
Postural instability (item 30, UPDRS III, scores ≥1, yes = 1)	2.66 (− 0.316, 5.64)	0.079	0.096
Global cognitive functioning (MoCA)	−0.374 (− 0.815, 0.066)	0.095	− 0.095
*Age*	*0.192 (0.037, 0.346)*	*0.015*	*0.144*
	R^2^ 63.0%; Adjusted R^2^ 61.4%		

Walk-12G = Generic Walk-12 (0–42, higher = worse); B = unstandardized regression coefficient; β = standardized regression coefficient; *FES-I* Falls Efficacy Scale-International (16–64, higher = worse), *UPDRS III* Unified Parkinson’s Disease Rating Scale, motor examination (item scores 0–4, higher = worse; those who scored ≥1 on item 30 were classified as having postural instability), *MoCA* Montreal Cognitive Assessment (0–30, higher = better)

^a^The following 13 independent variables were included in the initial model: sex; concerns about falling; perceived balance problems while dual tasking; postural instability; bradykinesia; freezing of gait; lower extremity function; orthostatism; pain; cognitive functioning; fatigue; general self-efficacy; depressive symptoms

*P*-values below 0.05 are bolded

Controlling factors are written in italic

#### Model 2 (controlled for age and baseline walk-12G scores)

The second model (controlled for age and baseline Walk-12G total scores) included six independent variables that predicted a change in perceived walking difficulties 3 years later, whereof two were statistically significant; the strongest predictor was perceived balance problems while dual tasking (β = 0.180) followed by global cognitive functioning (β = − 0.107). The remaining four variables in the model did not reach statistical significance: pain (*p* = 0.058), postural instability (*p* = 0.070), fatigue (*p* = 0.076) and lower extremity function (*p* = 0.099). The model accounted for 67.2% of the variance (adjusted R^2^) in Walk-12G scores at the 3-year follow-up. See Table 4 for further details.

**Table 4:** Multivariable linear regression analyses with Walk-12G at 3-year follow-up as the dependent variable: Model II (controlled for age and Walk-12G scores at baseline), *n* = 146

Independent variables (assessed at baseline)^a^	B (95% CI)	*P*-value	β
Dual task: perceived balance problems (yes = 1)	4.42 (1.55, 7.29)	**0.003**	0.180
Global cognitive functioning (MoCA)	−0.424 (− 0.830, − 0.017)	**0.041**	−0.107
Bothered by pain (yes = 1)	2.49 (− 0.087, 5.08)	0.058	0.100
Postural instability (item 30, UPDRS III, scores ≥1, yes = 1)	2.56 (−0.207, 5.33)	0.070	0.091
Fatigue (yes = 1)	2.44 (−0.261, 5.14)	0.076	0.101
Worse lower extremity function (Five chair stands test ≥16.0 s, yes = 1)^b^	− 2.13 (− 4.66, 0.403)	0.099	−0.088
*Age*	*0.233 (0.090, 0.375)*	*0.002*	*0.172*
*Perceived walking difficulties (Walk-12G), baseline*	*0.599 (0.445, 0.752)*	*< 0.001*	*0.529*
	R^2^ 69.0%; Adjusted R^2^ 67.2%		

Walk-12G = Generic Walk-12 (0–42, higher = worse); B = unstandardized regression coefficient; β = standardized regression coefficient; *MoCA* Montreal Cognitive Assessment (0–30, higher = better), *UPDRS III* Unified Parkinson’s Disease Rating Scale, motor examination (item scores 0–4, higher = worse; those who scored ≥1 on item 30 were classified as having postural instability)

Controlling factors are written in italic

*P*-values below 0.05 are bolded

^a^ The following 13 independent variables were included in the initial model: sex; concerns about falling; perceived balance problems while dual tasking; postural instability; bradykinesia; freezing of gait; lower extremity function; orthostatism; pain; cognitive functioning; fatigue; general self-efficacy; depressive symptoms

^b^ 12 participants did not manage the test. These were categorized as having worse lower extremity function (i.e. ≥ 16.0 s)

Both regression models fulfilled the assumptions for linear regression.

## Discussion

This is the first study that investigates how perceived walking difficulties evolve over time in people with PD, including identifying predictive factors. Our results showed that perceived walking difficulties increased significantly over 3 years. The first regression model (controlled for age) showed that concerns about falling was the strongest predictive factor for perceived walking difficulties, followed by perceived balance problems while dual tasking and being bothered by pain. The second model (controlled for age and baseline Walk-12G scores) showed that perceived balance problems while dual tasking was the strongest predictive factor for a change in perceived walking difficulties, followed by global cognitive functioning.

The mean Walk-12G score increased by 3.9 points over a 3-year period, which exceeds the measurement error (SEM) presented in this study as well as in a previous PD study [24]. The level of perceived walking difficulties in our sample at baseline seems to be in line with previous PD studies: the Walk-12G mean score was 14.8 in the present study as compared to 13–15.5 in previous studies [24, 32, 37]. However, one study reported a considerably lower Walk-12G score: median 8 (q1-q3, 4.5–21). This might be explained by a shorter PD duration (mean 5 years vs. median 8 years in the present study) and less motor symptoms (UPDRS III median 13 vs. 28 in the present study; data not presented) [38].

That concerns about falling was the strongest predictive factor of perceived walking difficulties is in line with prior cross-sectional studies, which described that fear of falling relates to walking difficulties in people with PD [32, 38]. In the current study, concerns about falling did not predict a change in perceived walking difficulties, i.e. when controlling for baseline Walk-12G scores. Controlling for a variable in a multivariable regression analysis means that the variable is included in all steps of the analysis, as well as in the final regression model. In the current study, FES-I scores were highly correlated with Walk-12G scores at baseline (r_s_ = 0.869), and this multicollinearity might explain why concerns about falling did not independently predict a change in perceived walking difficulties. Similarly, a previous study based on the same sample identified perceived walking difficulties as the strongest predictive factor of concerns about falling (FES-I), but it failed to predict a change in concerns about falling when controlling for baseline FES-I scores [22]. Perceived walking difficulties and concerns about falling are interconnected and adjacent aspects, but they are not interchangeable constructs. Perceived walking difficulties focus on problems connected to walking ability, whereas FES-I targets concerns about falling while performing 16 different activities. In FES-I, five out of the 16 items explicitly mention walking and an additional item addresses stairclimbing. The remaining 10 items address concerns about falling while for example getting dressed or undressed, preparing simple meals, reaching for something above your head or on the ground, visiting a friend or relative, and going out to a social event [30].

Perceived balance problems while dual tasking was the second strongest predictor of perceived walking difficulties, and it was the strongest predictor for a change in perceived walking difficulties. Prior studies showed that dual tasking negatively affects objective gait measures (e.g. gait speed, gait variability and gait rhythmicity) in people with PD [39, 40]. On the other hand, dual task training can be used to increase the level of difficulty when training gait and balance. Some studies suggest that dual task training has a positive effect on objective gait measures (e.g. dual task gait velocity, step length and cadence) in people with PD [41, 42]. Those who seems to benefit the most of dual task training are those with low initial gait speed when dual tasking and those with better cognitive functioning [41]. Interestingly, a study of highly challenging balance training, which included dual tasking, showed statistical significant improvements in balance performance and gait speed, but detected no improvements in any of the used patient reported outcomes, i.e. Walk-12G and rating scales targeting balance confidence and health-related quality of life [37]. Their findings might be due to that the intervention effect was considered clinically small, and the effect might therefore not transfer to perceived aspects such as Walk-12G scores. In other words, a potential explanation might be that objective changes in for example gait speed require large and/or long lasting changes in order to be reflected in patient-reported outcomes.

Pain showed to predict perceived walking difficulties, although it was less prominent for predicting a change in relation to baseline values (i.e. controlling for Walk-12G scores at baseline). Pain is a non-motor symptom that can affect up to 85% of people with PD, and it is most frequently located in the lower limbs [43]. Pain in people with PD can result in activity limitations such as walking difficulties [16, 44], and it has been shown to be negatively associated with quality of life [45]. In a prior cross-sectional study based on the same larger project as the present study (i.e. “Home and Health in People Ageing with Parkinson’s disease”), both pain and perceived walking difficulties were shown to be associated with decreased life-space mobility [46]. All considered, pain deserves attention at clinical follow-ups. Management of pain in people with PD may for example include medication, cognitive strategies or exercise [44, 47].

It needs to be noted that global cognitive functioning was included in both of the final models of the multivariable regression analyses. That is, better global cognitive functioning at baseline was associated with better perceived walking ability (i.e. less walking difficulties) 3 years later. Although this is a novel finding in relation to perceived walking difficulties, it is in line with prior studies that showed associations between cognitive functioning and objective gait difficulties in PD [13, 14, 48, 49]. Future studies are needed that address different cognitive domains in relation to perceived walking difficulties in people with PD.

Postural instability was included in both of the final regression models, although it failed to reach statistical significance. It was assessed by using item 30 of UPDRS part III, which is intended to assess the righting reflex and the reactive “response to a sudden posterior displacement produced by pull on shoulders” while standing erect with eyes open and feet slightly apart [27]. That both item 30 and perceived balance problems while dual tasking seems to be of importance for perceived walking difficulties highlight the importance of addressing balance problems in people with PD. Not the least since anti-PD medication insufficiently affects postural instability [50]. A meta-analysis found that physical exercise had a small positive effect on postural instability in people with PD, and “highly-challenging” balance training was advocated [51].

### Methodological considerations

At baseline, 243 participants completed the Walk-12G whereof 60.9% completed the questionnaire also at the 3-year follow-up. There are dropouts in all longitudinal studies, which can affect the external validity of the findings. We have previously reported that those who completed the assessments at both time points were significantly younger and had shorter PD-duration than those who were lost for follow-up [22].

Additional descriptive data might be valuable for this kind of study, such as the dose of anti-PD medications and other treatments that the participants might have had, e.g. rehabilitation and physical activity/exercise. Such data was not collected within this project, which is a shortcoming as it might affect how perceived walking difficulties evolve over time.

Twelve participants that did not manage the Five chair stands test were included in the group of participants categorized as having worse lower extremity function. However, there might be other reasons than poor lower extremity function that made them unable to complete the test. The categorization was done since excluding them might have rendered a final sample that was skewed towards having a better lower extremity functioning.

The independent variables in the present study were selected based on theoretical reasoning as well as on the results from previous cross-sectional studies [12, 20]. The regression models in our study explained 61.4 and 67.2% of the variance in the Walk-12G scores, respectively. This means that there are additional factors than those studied that can predict perceived walking difficulties. Future studies are needed to explore the effect of e.g. visual impairments on perceived walking difficulties in people with PD.

## Conclusions

Perceived walking difficulties increased over a 3-year period, and concerns about falling showed to be the strongest independent predictor. However, the latter was not the case when accounting for walking difficulties at baseline; perceived balance problems while dual tasking was then the strongest predictor. That is, both personal factors (i.e. concerns about falling) and motor aspects (i.e. balance problems while dual tasking) seem to play a role. Importantly, our study indicates that also non-motor symptoms (e.g. pain and cognitive functioning) seem to be of importance for future perceived walking difficulties.

Several of the identified factors are modifiable, and future intervention studies need to confirm whether addressing these factors have a preventative effect on perceived walking difficulties, i.e. as the primary outcome.

## Data Availability

The data used in this study contains sensitive information about the study participants and they did not provide consent for public data sharing. The current ethical approvals by the Regional Ethical Review Board in Lund, Sweden (No. 2012/558; 2015/611) do not include data sharing. A minimal data set could be shared by request from a qualified academic investigator for the sole purpose of replicating the present study, provided the data transfer is in agreement with EU legislation on the general data protection regulation and approval by the Swedish Ethical Review Authority. Contact information: Department of Health Sciences, Lund University Box 157, 221 00 Lund, Sweden DHSdataaccess@med.lu.se Principal investigator: Maria_H.Nilsson@med.lu.se Swedish Ethical Review Authority, Box 2110, 75 002 Uppsala, Sweden. Phone: + 46 10 475 08 00.

## Abbreviations

β: Standardized regression coefficients
B: Unstandardized regression coefficients
FES-I: Falls Efficacy Scale-International
FOG: Freezing of gait
FOGQsa: Self-administered version of the Freezing of Gait Questionnaire
GDS-15: Geriatric Depression Scale
GSE: General Self-Efficacy Scale
MoCA: Montreal Cognitive Assessment
NMSQuest: Non-Motor Symptoms Questionnaire
NHP-EN: Nottingham Health Profile, energy subscale
PD: Parkinson’s disease
SEM: Standard error of measurement
UPDRS: Unified Parkinson’s Disease Rating Scale
Walk-12G: Generic Walk-12
